# Model performance and interpretability of semi-supervised generative adversarial networks to predict oncogenic variants with unlabeled data

**DOI:** 10.1186/s12859-023-05141-2

**Published:** 2023-02-09

**Authors:** Zilin Ren, Quan Li, Kajia Cao, Marilyn M. Li, Yunyun Zhou, Kai Wang

**Affiliations:** 1 Raymond G. Perelman Center for Cellular and Molecular Therapeutics, Children’s Hospital of Philadelphia, Philadelphia, PA 19104 USA; 2 Princess Margaret Cancer Centre, University Health Network, University of Toronto, Toronto, ON M5G2C1 Canada; 3 Division of Genomic Diagnostics, Department of Pathology and Laboratory Medicine, Children’s Hospital of Philadelphia, Philadelphia, PA 19104 USA; 4 Department of Pathology and Laboratory Medicine, Perelman School of Medicine, University of Pennsylvania, Philadelphia, PA 19104 USA

**Keywords:** Generative adversarial networks, Variants annotation, Variants interpretation, Machine learning, Deep learning, Somatic variants

## Abstract

**Background:**

It remains an important challenge to predict the functional consequences or clinical impacts of genetic variants in human diseases, such as cancer. An increasing number of genetic variants in cancer have been discovered and documented in public databases such as COSMIC, but the vast majority of them have no functional or clinical annotations. Some databases, such as CiVIC are available with manual annotation of functional mutations, but the size of the database is small due to the use of human annotation. Since the unlabeled data (millions of variants) typically outnumber labeled data (thousands of variants), computational tools that take advantage of unlabeled data may improve prediction accuracy.

**Result:**

To leverage unlabeled data to predict functional importance of genetic variants, we introduced a method using semi-supervised generative adversarial networks (SGAN), incorporating features from both labeled and unlabeled data. Our SGAN model incorporated features from clinical guidelines and predictive scores from other computational tools. We also performed comparative analysis to study factors that influence prediction accuracy, such as using different algorithms, types of features, and training sample size, to provide more insights into variant prioritization. We found that SGAN can achieve competitive performances with small labeled training samples by incorporating unlabeled samples, which is a unique advantage compared to traditional machine learning methods. We also found that manually curated samples can achieve a more stable predictive performance than publicly available datasets.

**Conclusions:**

By incorporating much larger samples of unlabeled data, the SGAN method can improve the ability to detect novel oncogenic variants, compared to other machine-learning algorithms that use only labeled datasets. SGAN can be potentially used to predict the pathogenicity of more complex variants such as structural variants or non-coding variants, with the availability of more training samples and informative features.

**Supplementary Information:**

The online version contains supplementary material available at 10.1186/s12859-023-05141-2.

## Background

A large number of somatic variants have been identified by next-generation sequencing (NGS) in cancer research studies and clinical genetic testing laboratories. Except for recurrent “hotspot” mutations, clinical interpretation remains a significant challenge for many newly discovered variants. As of March 31, 2021, there are already 10 million variants curated in the COSMIC (Catalogue of Somatic Mutations in Cancer) database [1]. However, such “curation” only annotate somatic variant of genes from the CGC (Cancer Gene Census) [2], without knowing whether the variant is oncogenic or not. We refer to these variants as “unlabeled” variants, as we do not know their oncogenicity. On the other hand, a few existing databases contain manually labeled “oncogenic” variants, but these databases tend to be relatively small, because manual curations are costly ineffective. For example, the expert-curated database CIViC [3] only contains 2611 variants from 435 genes with literature-reported evidence in the current version (as of March 28^th^, 2021). Therefore, there is a stark contrast of “unlabeled” variants in public databases versus “labeled” variants for which clinical significance is already known and widely recognized. Given the rapid pace of discovering additional somatic variants from various types of cancer and the rapid expansion of COSMIC database, it is clear that the development of predictive tools is urgently needed to assess the clinical impacts of somatic variants in cancer.

Numerous tools for predicting the oncogenicity of cancer variants have been developed [4–13], using different types of genomic features and machine-learning (ML) algorithms, including deep learning (DL) algorithms. DL, as a sub-branch of ML, generally provides better performance with the availability of larger datasets in predictive tasks, compared to traditional ML methods. In feature selection, some of these existing tools used similar background information from alignment, evolutionary conservation, and homology, such as MutationAssessor[10], FATHMM-cancer[11], CHASM[5], and CanDrA[6]. In contrast, some other tools such as CTAT-cancer [13], used consensus features by integrating information from many other computational tools. In model selections, existing tools are only limited to supervised learning methods, even though they might use ensemble strategy to improve the predictive performance. For example, Agajanian et al. integrated several traditional ML approaches with deep convolutional neural networks (CNN) to improve the prediction of cancer variants [14]. Similarly, Wang et al. developed an ensemble ML method called AI-Driver to predict driver mutations based on 23 pathogenicity prediction scores [15]. They investigated the effects of feature selections and scaling methods, and evaluated the performance of supervised learning methods and pathogenicity scores.

However, there are several limitations of the existing tools for the prediction of oncogenic mutations. First, these tools typically do not incorporate clinical evidence (i.e., diagnosis, prognosis, etc.) as the predictive features, but only quantitative scores purely from computational predictions on effects of protein sequence, structure, conservation. Therefore, the prediction is more focused on prioritizing functionally important mutations instead of clinically important oncogenic mutations. Although the current tools achieved a better performance based on training and testing on several publicly available data sets, one major concern is that the data quality of public data resources is heterogeneous, and that they do not include clinical features. Second, the sample size of ‘labeled’ data from each data resource used for training is relatively small, in contrast to the millions of “unlabeled” somatic variants. The models trained on small labeled data using supervised ML learning methods may not achieve high predictive accuracy when applying to a large number of unlabeled samples. Several studies have shown that semi-supervised methods may improve predictive performance by incorporating “unlabeled” data in statistical models [16]; however, few studies applied this strategy to prioritize cancer somatic variants, to the best of our knowledge.

To address these limitations, in the current study, we developed a new semi-supervised generative adversarial neural network (SGAN) method, which incorporated 12 clinical features of somatic variants and unlabeled variants. Assuming all mutations, including oncogenic (driver) or passenger mutations, follow an underlying distribution, the SGAN method learns this underlying distribution by scanning all the possible variants from COSMIC and several other public knowledgebases. The 12 clinical evidence scores were derived from AMP/ASCO/CAP 2017 guideline, which is typically not used in previously published computational tools, to the best of our knowledge. Additionally, we also have access to ~ 6000 labeled variants that were manually labeled for their clinical significance by experts from a clinical diagnostic lab in our institute, which have a higher quality than public data sets. We comprehensively assessed the predictive performance of different algorithms, types of features, and training sample size in predicting cancer driver mutations to provide more insights in variant prioritization.

## Results

### Overview of the SGAN model for variant classification

**Fig. 1:**
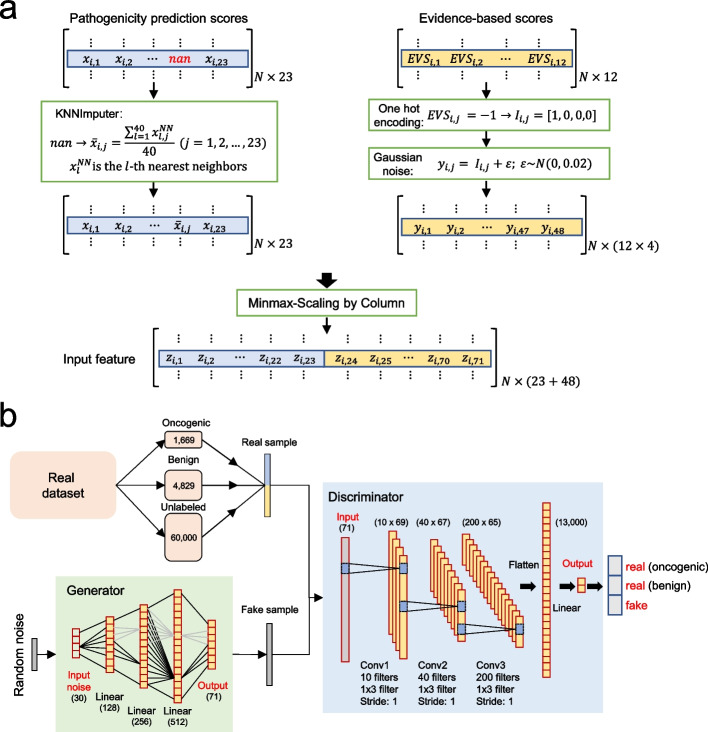
Schematic overview of SGAN method. **a** The SGAN method takes 23 prediction scores from available methods and 12 clinical evidence-based scores as input. Missing feature values were imputed with the mean of its 40 nearest neighboring variants. Discrete clinical evidence-based scores were converted into one-hot features adding Gaussian noise (mean is 0, standard deviation is 0.02) to make them continuous. Finally, the features were normalized by Minmax-scaling. **b** A total of 6498 labeled variants (1669 oncogenic and 4829 benign variants) and 60,000 unlabeled variants were used to train the model. The generative model generates fake (synthetic) samples by passing random noises into a linear model. The discriminative model distinguishes real samples from the fake, and classifies oncogenic variants and benign variants using labeled samples

An overview of the SGAN model for the prediction of oncogenic variants is shown in Fig. 1. Our study has three steps, including data preprocess, training semi-supervised learning model, and performance evaluation. Specifically, the labeled dataset used for training comprises 1669 oncogenic variants as positive (P) variants and 4892 benign variants as negative (N) variants. The labeled data for testing consists of 1335 oncogenic variants and 4829 neutral/benign variants that were manually collected from several experimentally annotated studies in PubMed [17–21]. For the unlabeled dataset, we collected ~ 13 million exonic variants in 1685 genes from several existing cancer knowledgebases (see Methods for details). We used multiple performance measures, including precision, recall, F1 score, Matthew’s correlation coefficient (MCC) and others. To evaluate how the model performance changes with respect to model parameterization, we also tested the effects of changing input data size, performing feature selection, and compared the performance of SGAN to pure supervised learning (without unlabeled data) algorithms and other computational tools.

### Interpretability of the SGAN model for variant interpretation

**Fig. 2:**
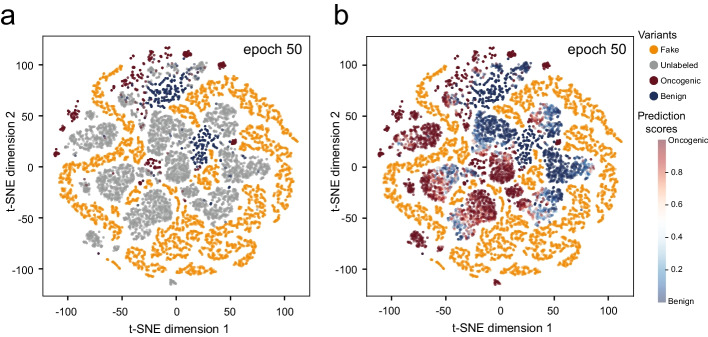
T-SNE plot of the training data set, includng 10,000 fake variants from the trained generator (training epoch is 50), 10,000 unlabeled variants and 2000 labeled variants (1000 oncogenic and 1000 benign variants) from an in-house database of expert-annotated somatic variants. **a** Gery data points are unlabeled variants. **b** Unlabeled variants with predicted interpretation scores are ranging from 0 to 1. And variants with high interpretation scores (close to red) are predicted as oncogenic

To gain insights into how SGAN works, we employed a two-dimensional t-distributed stochastic neighbor embedding (t-SNE) [22] for visualization (Fig. 2). In principle, the G (generator) was trained to generate fake datapoints following the underlying distribution of real data. If there was any difference between the real and fake (synthetic) datapoints, the D (discriminator) would distinguish them. When the G fools the D, it means that the G has learnt the underlying distribution, and then the D will give clear boundaries among data groups. Then, the unlabeled data would be aggregated into several clusters and predicted to be the same categories as their adjacent labeled data. In Fig. 2, there are 10,000 fake variants (orange data points), 10,000 real unlabeled data points (grey data points), 2000 real labeled variants (dark red for oncogenic and dark blue for benign) for t-SNE plotting. As we can see in Fig. 2a, fake variants could be regarded as boundaries of clusters providing information for classifying unlabeled data. To visualize the predictive interpretability, we used gradient colors to display the SGAN predicted scores for each variant (Fig. 2b). The interpretation score is the probability of oncogenicity for variants ranging from 0 to 1. A variant with a higher interpretation score will be more likely to be classified as oncogenic by SGAN. According to the prediction result, we found that the unlabeled datapoints with high interpretation scores were close to labeled oncogenic variants, which is consistent with the truth. Several additional clusters can be seen in the t-SNE figure, which should include both oncogenic and neutral variants, but we could still find clear boundaries between these clusters. In summary, this analysis indicated that both boundaries of fake data points and categories of labeled data points are considered to make the prediction, which is what SGAN model is trying to achieve during the training process, by the generator and discriminator functions.

### Performance comparison when using different sample sizes of labeled data

**Fig. 3:**
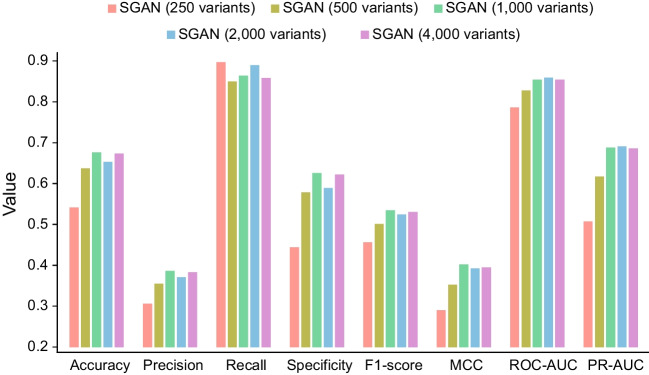
Evaluation of the performance of SGAN models based on different number of labeled variants being used in training process. We used 250, 500, 1000, 2000, and 4000 variants in supervised training process when training SGAN models

To evaluate how the size of labeled samples in the training set influences the prediction accuracy of unlabeled data, we trained model with a different number of labeled variants, ranging from 250 to 4000 as mentioned in Methods. For each variant in the testing dataset, we calculated the interpretation scores using the Softmax function. We used interpretation scores for ROC AUC and PR AUC comparison. For the rest of the comparison metrics, such as accuracy, F1, and MCC, we used interpretation score > 0.5 as the cut-off to determine whether a variant is oncogenic or neutral for equal comparison. All eight-evaluation metrics were summarized into a barplot (Fig. 3) and a table (Additional file 1 Table S2). As we can see from the results, the performance would be generally improved when the size of the labeled dataset increased. In particular, when the number of labeled data increased from 250 to 1000, the MCC increased from 0.29 to 0.402 and the PR-ROC increased from 0.507 to 0.688. However, what makes the SGAN prediction intriguing is that when the number of labeled datapoints exceeds 1000, the performance of the SGAN model appeared to be flattened. It may suggest that SGAN model can achieve comparable performance with smaller training samples, as long as labeled data shares similar distribution with unlabeled data (see Fig. 2), which is the unique advantage of the SGAN method. We also summarized the performance metrics with 10 different cutoffs (ranging from 0.1 to 0.9, and 0.95). The result showed that when the labeled samples reached 4000 and when the cutoff was 0.95, the accuracy reached 0.853 and the MCC reached 0.538.

### Performance comparison for different types of features

To investigate how predictive features influence the prediction accuracy, we trained three models with different types of features as the input, including: (1) pathogenicity prediction scores, (2) evidence-based scores, and (3) ensemble scores. The performance metrics have been summarized in Table 1. Compared to models using pathogenicity prediction scores only, the performance of model using evidence-based scores was better in all metrics, except for sensitivity when the number of labeled variants was under 1000. We believe that the evidence-based scores provide more insights for oncogenicity prediction than scores obtained from current pathogenicity prediction tools. Although 6 of 8 performance metrics of using evidence-based scores with 4000 labeled variants were the best in all cases, the performance of ensemble scores showed many improvements on variant interpretation according to ROC-AUC and PR-AUC values (ROC-AUC: 0.859, PR-AUC: 0.691), and the other six metric values depend highly on the choice of threshold to be oncogenic. Therefore, it is essential to use both pathogenicity prediction scores and evidence-based scores for model construction.

**Table 1:** Performance comparison when using different number of labeled variants and different types of features. We trained SGAN models based on different size of labels (ranging from 250 to 4000 variants) and three groups of features: pathogenicity prediction scores, evidence-based scores, and ensemble scores (full features)

Features	Training size	Accuracy	Precision	Recall	Specificity	F1score	MCC	ROC AUC	PR AUC
Pathogenicity prediction scores	250	0.631	0.339	0.757	0.597	0.468	0.29	0.732	0.413
	500	0.643	0.346	0.748	0.614	0.473	0.299	0.741	0.421
	1000	0.625	0.337	0.774	0.584	0.469	0.294	0.734	0.412
	2000	0.642	0.346	0.751	0.613	0.474	0.299	0.737	0.413
	4000	0.631	0.34	0.767	0.594	0.471	0.297	0.734	0.406
Evidence-based scores	250	0.727	0.415	0.667	0.743	0.512	0.355	0.815	0.543
	500	0.75	0.447	0.706	0.762	0.548	0.406	0.814	0.627
	1000	0.729	0.428	0.778	0.716	0.552	0.416	0.848	0.676
	2000	0.705	0.406	0.81	0.677	0.541	0.404	0.849	0.678
	4000	**0.78**	**0.492**	0.781	**0.78**	**0.604**	**0.486**	0.832	0.647
Ensemble	250	0.541	0.306	**0.897**	0.444	0.456	0.29	0.786	0.507
	500	0.637	0.355	0.85	0.579	0.501	0.352	0.828	0.617
	1000	0.676	0.386	0.864	0.625	0.534	0.402	0.854	0.688
	2000	0.653	0.371	0.889	0.589	0.524	0.392	**0.859**	**0.691**
	4000	0.673	0.383	0.858	0.622	0.53	0.395	0.854	0.686

### Performance comparison for different types of tools and ML algorithms

**Fig. 4:**
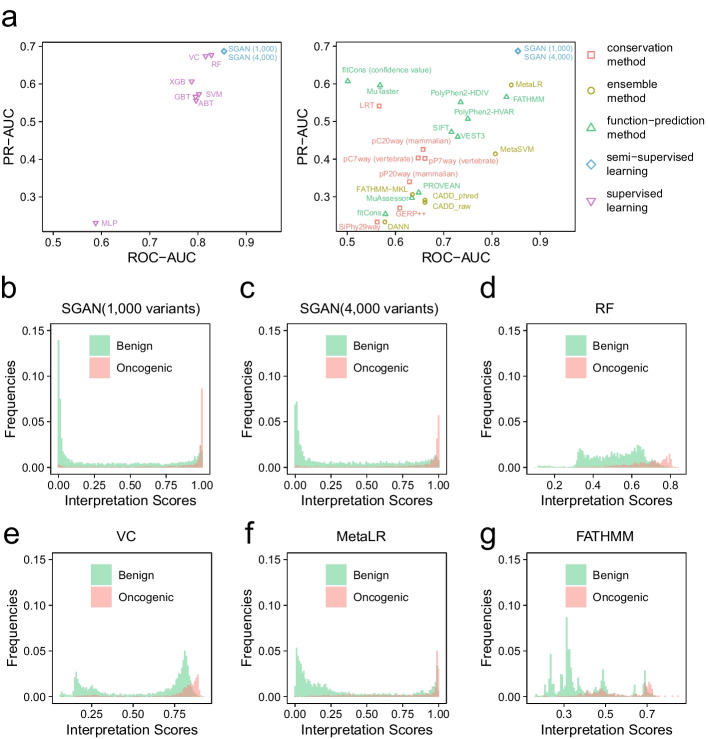
Performance comparison among different methods. **a** PR vs. ROC AUC plot for SGAN models, machine-learning models (left panel), and 23 in silico algorithms or tools (right panel). The shapes and colors represent the types of these methods. **b**–**g** Distribution of interpretation scores for somatic mutations in the testing dataset. The interpretation scores were predicted by **b** SGAN model using 1000 labeled variants, **c** SGAN model using 4000 labeled variants, **d** random forest model, **e** voting classifier, **f** MetaLR, and **g** FATHMM

Compared with supervised learning methods and existing pathogenicity prediction methods, the SGAN models achieved the best performance of discriminating oncogenic and benign variants according to ROC-AUC and PR-AUC values (Fig. 4a and Additional file 1 Table S3). With 1000 labeled variants, the ROC-AUC score of SGAN was 0.854 and PR-AUC score was 0.688. For supervised learning, ROC-AUC scores ranged from 0.588 (MLP) to 0.828 (RF), and PR-AUC scores ranged from 0.231 (MLP) to 0.677 (RF). For other prediction tools, metaLR (ROC-AUC was 0.84 and PR-AUC was 0.597) and FATHMM (ROC-AUC was 0.83 and PR-AUC was 0.565), SGAN also outperformed other methods. To get insight into interpretation scores predicted by methods, we generated histogram of interpretation scores. For the SGAN method, the oncogenic scores were mainly close to 1 (Fig. 4b-c). However, some false positive variants might exist, which can be solved by increasing the number of labeled variants. For supervised learning, the interpretation scores of oncogenic variants predicted by RF were mainly in the range of 0.7 and 0.8 (Fig. 4d), and the interpretation scores for those neutral variants were evenly distributed between 0.2 and 0.7. The interpretation scores obtained by VC indicated an overfitting problem, since the peak of benign interpretation scores was very close to the peak of oncogenic interpretation scores (Fig. 4e). The distribution of interpretation scores predicted by MetaLR is similar to SGAN (Fig. 4f), but there might be more false positives. For FATHMM, there was too much overlap between the interpretation scores of oncogenic and neutral variants (Fig. 4g). In summary, we found that the SGAN model was easy to identify the oncogenic variants with fewer labeled variants during the training process than other methods.

### Performance comparison for the prediction of loss of function (LoF) and gain of function (GoF) mutations

We compared the prediction of driver LoF and GoF mutations with statistically significantly recurrent mutations identified in large scale cancer genomics data reported in CancerHotspot (https://www.cancerhotspots.org). According to Bozic et al.’s. theoretical estimation, the number of GoF in one oncogene is supposed to be fewer than LoF in one tumor suppressor gene[23]. In total, we collected 59 GoF mutations from 10 oncogenes and 1375 LoF mutations from 10 tumor suppressor genes in the CancerHotspot database. We found that 48 of 59 GoF mutations and 771 of 1375 LoF mutations were predicted as driver mutations with the cutoff of 0.95. The predictive score distribution for GoF and LoF are different, which can be found in Additional file 1 Table S4.

## Discussion

Previous WES/WGS studies identified millions of somatic mutations from a broad range of cancer types. However, our knowledge of distinguishing oncogenic mutations from neutral ones remains limited. Therefore, the majority of the somatic mutations were classified as variants of unknown significance (VUS). The presence of many VUS greatly impeded the effectiveness of clinical management of patients with cancer. For example, there are sporadic reports that doctors recommend bilateral mastectomies to patients with suspected P/LP in *TP53* or *BRCA1*, which later turned out to be benign [24]. This critical knowledge gap leads to key challenges in implementing precision medicine to guide optimal treatment strategies. To generate a prioritized list for variants with clinical impacts, in this study, we introduced a novel semi-supervised method called SGAN, incorporating a combination of feature metrics from both human-defined rule-based scoring metrics and predictive model-based scoring metrics.

The main rationale for using the semi-supervised GAN algorithm here is that the number of labeled variants is typically small, resulting in a computational challenge in learning from small samples in the real world; yet, by incorporating unlabeled data into a semi-supervised GAN model, the training procedure can be greatly improved. One unique advantage of semi-supervised GAN is that it has less overfitting issues when learning from small samples, due to the property of underlying distribution assumption of the data modeling in GAN. Traditional GAN model consists of 2 parts: generator to generate synthetic (fake) samples, and discriminator to classify samples as either real or fake. But here, we improved traditional GAN by including labeled samples, unlabeled samples, and fake samples that were generated by the generator. The generator’s goal is to generate samples similar to the real samples as much as possible in the learning process. This network has the unique advantage in dynamically learning the underlying distribution (clusters) of data samples by discriminating the synthetic samples and unlabeled real samples at each epoch. In principle, due to the advantage of the unique architecture, SGAN can be potentially used to predict more complex variants such as structural variants or non-coding variants with some modifications, considering they are typically suffered from the limited number of ‘labeled’ samples as the training set.

SGAN uses a small amount of labeled data and a large amount of unlabeled data, which provides the benefits of both unsupervised and supervised learning while avoiding the challenges of finding a large amount of labeled data. Our results showed that, even with 1000 labeled samples, the prediction performance of SGAN is as good as using 4000 samples in the training set. Another major challenge to differentiate oncogenic variants from VUS accurately is that even within the small number of labeled samples, positive (oncogenic) and negative (neutral) classes are unbalanced. We overcome this issue through resampling technique during the training process. Additionally, many previously published methods use only deleteriousness prediction scores in machine-learning models, without considering evidence-based scores typically defined in clinical guidelines. In this project, our SGAN method overcomes these challenges, using both evidence-based and model-based score features, many of which are not used in existing tools, to achieve the best predictive performance.

Although SGAN can accelerate the clinical interpretation process on cancer variants, our model-based approach cannot replace human reviewers. We need to explore more mutations with reliable labels manually to improve cancer diagnosis. We stress that our method has the following limitations: First, the number of testing datasets is limited, and we only used somatic mutations, which are not comprehensive enough to evaluate the performance of our model, and the best cutoff for determining oncogenicity probably needs to be adjusted on a case by case basis in real studies, depending on the data distribution. Second, the current SGAN model cannot interpret complicated genomic variants, such as inversions and gene fusions, and cannot interpret gene expression alterations, even though these genomic alterations may also play important roles in tumorigenesis and cancer progression. In principle, all these mutations can be used to build a new SGAN model with a different set of features, and there are already many unlabeled data in COSMIC. However, the major challenge here is that we may not have a sufficient amount of labeled data and a sufficient number of predictive features to train a reliable model. Third, we did not consider coding indels (especially frameshift indels which results in premature stop codons), because their clinical interpretations are generally straightforward in existing clinical guidelines. Nevertheless, we do acknowledge that many computational tools are developed for predicting functional significance of coding indels, in addition to SNPs, so we may explore the possibility of building a SGAN for these indels later. Additionally, we also acknowledge that a small fraction of non-coding variants may be highly penetrant to be oncogenic; however, such validation data is extremely sparse. Finally, we acknowledge that non-canonical splice variants may be oncogenic and can be interpreted by current clinical guidelines, but they do not have feature scores similar to missense variants. We will explore the use of several splice variants prediction algorithms (such as SpliceFinder [25], SPIDEX [26], dbscSNV [27]) as additional features in the SGAN model for these variants in the future.

## Conclusion

By incorporating many large samples of unlabeled data, the SGAN method can improve the ability to detect novel oncogenic variants, compared to other machine-learning algorithms that use only labeled datasets. SGAN can be potentially used to predict the pathogenicity of more complex variants such as structural variants or non-coding variants, with the availability of more training samples and informative features.

## Methods

### Datasets

We collected three types of datasets: one labeled dataset and one unlabeled dataset were used for training and validation, while the third labeled dataset was used for testing. For the labeled dataset, we have 6498 expert-curated variants from cancer patients in an in-house database as the training set, and 6164 variants from public resources as the testing set. Specifically, the labeled dataset used for training comprises 1669 oncogenic variants as positive (P) variants and 4829 benign variants as negative (N) variants. The labeled data for testing consists of 1335 oncogenic variants and 4829 neutral/benign variants that were manually collected from several experimentally annotated studies in PubMed [17–21]. For the unlabeled dataset, we collected ~ 13 million exonic variants in 1685 genes from 7 existing cancer knowledgebases, including OncoKB[28], Cosmic[1], Cancer Genome Interpreter (CGI) [29], IntoGen [30], CIViC [3], JAX-Clinical Knowledgebase (CKB) [31], and Precision Medicine Knowledge Base (PMKB) [32]; as well as two datasets about driver genes predictions published by Bailey et al. [33] and Dietlein et al. [34].

### Predictive features

Two types of predictive features were used to generate score metrics for the model: clinical scores and functional deleteriousness scores. First, 12 clinical evidence-based prediction scores according to the AMP/ASCO/CAP 2017 guideline [35] were obtained from CancerVar [36], which is an automated evidence collection tool recently developed by our group. Because the clinical evidence-based prediction scores are discrete variables (−1: benign; 0: no support; 1: supporting clinical significance or oncogenic; 2: strong clinical significance evidence or oncogenic), we converted them into dummy features by adding Gaussian noise (mean = 0, SD = 0.02), which made them continuous within a small range. Second, 23 deleteriousness scores were obtained from ANNOVAR [37] through dbNSFP database [38], including (1) nine function-prediction method: FATHMM[11], FitCons [39], MutationAssessor [10], Mutation Taster [40], PolyPhen2-HDIV [41], PolyPhen2-HVAR [41], PROVEAN [42], SIFT [43], and VEST3 [44]; (2) five ensemble methods: CADD (raw score and Phred score) [45], DANN [46], FATHMM-MKL [47], MetaLR [48], and MetaSVM [48]; and (3) five conservation methods: GERP +  + [49], PhastCons [50] (on vertebrate and mammalian separately), PhyloP [51] (on vertebrate and mammalian separately), LRT [52], and SiPhy [53]. We first arbitrarily removed the variants that have more than 13 missing values for missing values in features. And then, we implemented the KNNImputer, a python tool from scikit-learn toolkit [54], to impute the missing values for features with the mean of its 40 nearest neighboring variants. Finally, scores for labeled and unlabeled samples were normalized by Min–Max scaling. Data preprocessing workflow is shown in Fig. 1a.

### Overview of the semi-supervised learning model for variant interpretation

Our model is an improved version of semi-supervised GAN. In detail, the SGAN contains 2 parts: (1) generator (G), which generates synthetic observations with a vector of Gaussian noise as input; (2) discriminator (D), which determines whether the observation is synthetic or real in unsupervised training process, and to classify whether the observation is oncogenic or benign in supervised training process. The generator consists of 4 linear layers, with LeakyReLU as the activation layer and Tanh as the last activation layer before output. We also used batch normalization after each linear layer and a dropout rate of 0.6 in hidden layers. For the discriminator, we used a 3-layer convolutional neural network, which is shown in Fig. 1b.

In detail, assuming z is a vector of Gaussian noise, a perfectly trained generator network $G(z, {\theta }^{(G)})$ can produce a sample following the real data distribution ${p}_{data}\left(x\right)$. In other words, the generator can learn real data distribution, even without any label. And the discriminator network $D(z, {\theta }^{(D)})$ is trained to distinguish samples from the generator distribution from real data. The groups of real datapoints are labeled as oncogenic groups or benign groups, and the datapoints that exceed the group boundary are considered as synthetic, the discriminator constantly refines the boundaries of those groups (oncogenic, benign, and synthetic groups) in the unsupervised training. However, the discriminator has the ability of labeling the groups with oncogenic or benign using a small number of labeled datapoints in supervised training.

In our SGAN model training process, we first trained the discriminator and then the generator in each minibatch. We trained the discriminator by minimizing the sum of supervised learning loss and unsupervised learning loss. For the supervised learning, the discriminator, working as a standard classifier, takes the labeled data point x as input and outputs a 2-dimenisonal vector ${l}_{1},{l}_{2}$. Then, the class probabilities (benign or oncogenic) by the softmax function can be written as:

$$p_{model} {(}y = i {|}x) = \frac{{exp\left( {l_{i} } \right)}}{{exp\left( {l_{1} } \right) + exp\left( {l_{2} } \right)}},i = 1,2.$$

Therefore, binary cross entropy between the true labels and the model predictive distribution ${p}_{model}\left(y \right|x)$ was obtained as the supervised learning loss:

$$L_{supervised} = - E_{{x,y\sim p_{{data\left( {x,y} \right)}} }} logp_{model} \left( {y = i{|}x,y} \right), \; where \; i = 1 \; or \; 2.$$

For the unsupervised learning, we labeled the data point G(z) with “synthetic” and used ${p}_{model}$ (y = 3| x) to indicate the probability that the sample is from the generator. Based on the property of method [1, 2], we don’t need to add a new dimension of output in the discriminator. The probability for real or synthetic is written like following:

$$D\left( x \right) = \frac{Z\left( x \right)}{{Z\left( x \right) + 1}}, \; where \; Z\left( x \right) = exp\left( {l_{1} } \right) + exp\left( {l_{2} } \right),$$

where x is an unlabeled data point and ${l}_{1},{l}_{2}$ are the logits from the final layer of the discriminator mentioned above. For a real unlabeled data point x, whether it is benign or oncogenic, the value of $Z\left(x\right)$ will be very large, and then the value of $D\left(x\right)$ will be close to 1. Otherwise, the value of D(x) will be close to 0 if ${l}_{1},{l}_{2}$ are small. Therefore, the unsupervised learning loss and the loss of the discriminator can be written as:

$$L_{unsupervised} = - \left\{ {E_{{x\sim p_{data\left( x \right)} }} logD\left( x \right) + E_{z\sim noise} \log \left( {1 - D\left( {G\left( z \right)} \right)} \right)} \right\}$$

$$L = L_{supervised} + L_{unsupervised}$$

And the generator (G) was trained by minimizing feature matching loss, which is referred to Tim et al. [55]

$$\left\| {E_{{x\sim p_{data} }} D\left( x \right) - E_{z\sim noise} D\left( {G\left( z \right)} \right)} \right\|_{2}^{2}$$

We used Adam optimizer [56] with an initial learning rate of 0.0095 to minimize the discriminator and generator loss separately.

### Overview of machine-learning approaches used for performance comparison.

For comparison purpose, we employed seven supervised machine-learning methods, which were provided in a recently published paper AI-Driver [15], including support vector machine (SVM), random forest (RF), adaptive boosting (AdaBoost), gradient tree boosting (GBT), voting classifier (VC), multi-layer perceptron (MLP), and eXtreme Gradient Boosting (XGBT). In AI-Driver, input data is vectors composed of PHRED-scaled pathogenic prediction scores and the performance of models was slightly better than those of using pathogenic prediction scores without any preprocessing. Besides the supervised learning method mentioned above, we also investigated 23 features with pathogenic prediction scores.

### Training process

To perform a comprehensive assessment on semi-supervised learning for variant interpretation, we trained five models with different number of labeled variants, ranging from 250 to 4000 (Additional file 1: Table S1). Meantime, we randomly selected 60,000 unlabeled variants from the unlabeled dataset as input for unsupervised learning. In each minibatch, 500 labeled variants, 500 unlabeled variants, and 500 synthetic variants were used to train the discriminator and the generator separately. The whole process was built with PyTorch 1.6.0 and Python 3.8.4 [57]. It took ~ 5 h to train 2000 epochs on NVIDIA GeForce GTX 1080Ti.

We randomly selected 4000 variants (1000 oncogenic variants P and 3000 benign variants N) from the training dataset for machine-learning methods. The rest variants (669 P and 565 N variants) in the training dataset were used for validation. Function prediction scores of these variants were obtained from dbnsfp30a using ANNOVAR (version 2020-06-07).

### Evaluation metrics

We used 8 performance measurements for performance comparison, including accuracy, precision, sensitivity(recall), specificity, F1 score, Matthew’s correlation coefficient (MCC), area under the receiver operating characteristic curve (ROC-AUC), and area under the Precision-Recall Curve (PR-AUC). The number of true positive predictions is denoted as TP (false positive for FP, true negative for TN, and false negative for FN). Thus, we computed the accuracy, precision, recall, specificity, F1 score, and MCC as

$$accuracy = \frac{TP + TN}{{TP + TN + FP + FN}}$$

$$precision = \frac{TP}{{TP + FP}}$$

$$Sensitivity = \frac{TP}{{TP + FN}}$$

$$specificity = \frac{TN}{{FP + TN}}$$

$$F1\;score = \frac{{2 \times \left( {precision \times recall} \right)}}{{\left( {precision + recall} \right)}}$$

$$MCC = \frac{TP \times TN - TP \times FN}{{\sqrt {\left( {TN + FN} \right) \times \left( {TN + FP} \right) \times \left( {TP + FN} \right) \times \left( {TP + FP} \right)} }}$$

MCC is a measurement of the quality of binary classifier, ranging from -1 to 1. Compared to F1 scores, it overcomes the issue of class imbalance in evaluation studies. A coefficient of -1 indicates that prediction is inconsistent with observation, 0 means like random prediction, and 1 for a perfect classifier. Considering that MCC value depends on the cutoff of binary classifier, we also calculated ROC-AUC and PR-AUC values for model comparisons.

## Supplementary Information

**Additional file 1: Table S1**. The number of variants used in training process**Table S2**. Performance of SGAN with different determination thresholds and different threshold**Table S3**. Performance comparison among different methodsTable S4. Predictive score distribution for loss of function mutations and gain of function mutations.

## Data Availability

The code generated and datasets analyzed during the current study are available in the GitHub repository: https://github.com/WGLab/SGAN.

## Abbreviations

SGAN: Semi-supervised generative adversarial networks
CNN: Convolutional neural networks
MCC: Matthew’s correlation coefficient
ROC-AUC: Area under the receiver operating characteristic curve
PR-AUC: Area under the Precision-Recall Curve
SVM: Support vector machine
RF: Random forest
AdaBoost: Adaptive boosting
GBT: Gradient tree boosting
VC: Voting classifier
MLP: Multi-layer perceptron
XGBT: EXtreme Gradient Boosting
