# Correction: Model performance and interpretability of semi-supervised generative adversarial networks to predict oncogenic variants with unlabeled data

**DOI:** 10.1186/s12859-023-05357-2

**Published:** 2023-05-31

**Authors:** Zilin Ren, Quan Li, Kajia Cao, Marilyn M. Li, Yunyun Zhou, Kai Wang

**Affiliations:** 1 Raymond G. Perelman Center for Cellular and Molecular Therapeutics, Children’s Hospital of Philadelphia, Philadelphia, PA 19104 USA; 2 Princess Margaret Cancer Centre, University Health Network, University of Toronto, Toronto, ON M5G2C1 Canada; 3 Division of Genomic Diagnostics, Department of Pathology and Laboratory Medicine, Children’s Hospital of Philadelphia, Philadelphia, PA 19104 USA; 4 Department of Pathology and Laboratory Medicine, Perelman School of Medicine, University of Pennsylvania, Philadelphia, PA 19104 USA

**Correction : BMC Bioinformatics (2023) 24:43** **https://doi.org/10.1186/s12859-023-05141-2**

Following publication of the original article [1], it was reported that the article entitled “Model performance and interpretability of semi-supervised generative adversarial networks to predict oncogenic variants with unlabeled data” was published in the regular issue of this journal instead of in the supplement issue.

The details of the supplement in which this article ought to have been published are given below:

**About this supplement**

This article has been published as part of BMC Bioinformatics Volume 23 Supplement 3, 2022: Selected articles from the International Conference on Intelligent Biology and Medicine (ICIBM 2021): bioinformatics. The full contents of the supplement are available online at https://bmcbioinformatics.biomedcentral.com/articles/supplements/volume-23-supplement-3.

The publisher apologizes for any inconvenience caused.
