# Supplementary material for: Model performance and interpretability of semi-supervised generative adversarial networks to predict oncogenic variants with unlabeled data
Source: BMC Bioinformatics. 2023 Feb 9;24:43. doi: 10.1186/s12859-023-05141-2 (PMC9909865; doi:10.1186/s12859-023-05141-2)
Supplement: Supplementary file 1 — Additional file 1: Table S1. The number of variants used in training processTable S2. Performance of SGAN with different determination thresholds and different thresholdTable S3. Performance comparison among different methodsTable S4. Predictive score distribution for loss of function mutations and gain of function mutations. [file 12859_2023_5141_MOESM1_ESM.docx]

**Supplementary table 1.** The number of variants used in training process.

| **training size** | **No. of oncogenic variants** | **No. of benign variants** | **No. of unlabeled variants** |
| --- | --- | --- | --- |
| 250 | 50 | 200 | 60,000 |
| 500 | 100 | 400 | 60,000 |
| 1,000 | 250 | 750 | 60,000 |
| 2,000 | 500 | 1,500 | 60,000 |
| 4,000 | 1,000 | 3,000 | 60,000 |

**Supplementary table 2.** Performance of SGAN with different determination thresholds and different threshold.

| **label size** | **threshold** | **Accuracy** | **Precision** | **Recall** | **Specificity** | **F1score** | **MCC** | **ROC-AUC** | **PR-AUC** |
| --- | --- | --- | --- | --- | --- | --- | --- | --- | --- |
| 250 | 0.1 | 0.484 | 0.286 | 0.939 | 0.36 | 0.438 | 0.268 | 0.786 | 0.507 |
|  | 0.2 | 0.508 | 0.294 | 0.928 | 0.393 | 0.447 | 0.281 |  |  |
|  | 0.3 | 0.52 | 0.299 | 0.919 | 0.411 | 0.451 | 0.286 |  |  |
|  | 0.4 | 0.531 | 0.302 | 0.906 | 0.429 | 0.453 | 0.287 |  |  |
|  | 0.5 | 0.541 | 0.306 | 0.897 | 0.444 | 0.456 | 0.29 |  |  |
|  | 0.6 | 0.555 | 0.311 | 0.884 | 0.466 | 0.46 | 0.294 |  |  |
|  | 0.7 | 0.569 | 0.314 | 0.855 | 0.491 | 0.46 | 0.288 |  |  |
|  | 0.8 | 0.597 | 0.326 | 0.825 | 0.534 | 0.467 | 0.296 |  |  |
|  | 0.9 | 0.643 | 0.349 | 0.766 | 0.609 | 0.479 | 0.309 |  |  |
|  | 0.95 | 0.697 | 0.388 | 0.711 | 0.694 | 0.502 | 0.34 |  |  |
| 500 | 0.1 | 0.536 | 0.306 | 0.917 | 0.432 | 0.459 | 0.299 | 0.828 | 0.617 |
|  | 0.2 | 0.563 | 0.317 | 0.9 | 0.471 | 0.469 | 0.312 |  |  |
|  | 0.3 | 0.582 | 0.326 | 0.889 | 0.498 | 0.477 | 0.323 |  |  |
|  | 0.4 | 0.609 | 0.339 | 0.871 | 0.537 | 0.488 | 0.337 |  |  |
|  | 0.5 | 0.637 | 0.355 | 0.85 | 0.579 | 0.501 | 0.352 |  |  |
|  | 0.6 | 0.671 | 0.378 | 0.831 | 0.627 | 0.52 | 0.377 |  |  |
|  | 0.7 | 0.708 | 0.408 | 0.803 | 0.682 | 0.541 | 0.403 |  |  |
|  | 0.8 | 0.751 | 0.451 | 0.748 | 0.752 | 0.563 | 0.428 |  |  |
|  | 0.9 | 0.799 | 0.524 | 0.655 | 0.838 | 0.582 | 0.457 |  |  |
|  | 0.95 | 0.826 | 0.596 | 0.588 | 0.891 | 0.592 | 0.482 |  |  |
| 1,000 | 0.1 | 0.528 | 0.305 | 0.94 | 0.415 | 0.461 | 0.308 | 0.854 | 0.688 |
|  | 0.2 | 0.57 | 0.323 | 0.921 | 0.474 | 0.479 | 0.333 |  |  |
|  | 0.3 | 0.605 | 0.34 | 0.896 | 0.525 | 0.493 | 0.349 |  |  |
|  | 0.4 | 0.641 | 0.361 | 0.882 | 0.575 | 0.513 | 0.375 |  |  |
|  | 0.5 | 0.676 | 0.386 | 0.864 | 0.625 | 0.534 | 0.402 |  |  |
|  | 0.6 | 0.713 | 0.417 | 0.841 | 0.679 | 0.557 | 0.431 |  |  |
|  | 0.7 | 0.751 | 0.455 | 0.813 | 0.734 | 0.583 | 0.462 |  |  |
|  | 0.8 | 0.776 | 0.486 | 0.77 | 0.778 | 0.596 | 0.475 |  |  |
|  | 0.9 | 0.814 | 0.553 | 0.7 | 0.845 | 0.618 | 0.503 |  |  |
|  | 0.95 | 0.836 | 0.615 | 0.631 | 0.892 | 0.623 | 0.519 |  |  |
| 2,000 | 0.1 | 0.47 | 0.283 | 0.962 | 0.335 | 0.437 | 0.274 | 0.859 | 0.691 |
|  | 0.2 | 0.523 | 0.303 | 0.942 | 0.409 | 0.458 | 0.305 |  |  |
|  | 0.3 | 0.57 | 0.323 | 0.919 | 0.474 | 0.478 | 0.331 |  |  |
|  | 0.4 | 0.607 | 0.342 | 0.902 | 0.526 | 0.496 | 0.355 |  |  |
|  | 0.5 | 0.653 | 0.371 | 0.889 | 0.589 | 0.524 | 0.392 |  |  |
|  | 0.6 | 0.7 | 0.406 | 0.864 | 0.655 | 0.552 | 0.427 |  |  |
|  | 0.7 | 0.743 | 0.446 | 0.826 | 0.72 | 0.579 | 0.458 |  |  |
|  | 0.8 | 0.779 | 0.49 | 0.778 | 0.779 | 0.601 | 0.482 |  |  |
|  | 0.9 | 0.817 | 0.559 | 0.694 | 0.851 | 0.619 | 0.505 |  |  |
|  | 0.95 | 0.845 | 0.646 | 0.613 | 0.908 | 0.63 | 0.532 |  |  |
| 4,000 | 0.1 | 0.494 | 0.291 | 0.949 | 0.369 | 0.446 | 0.285 | 0.854 | 0.686 |
|  | 0.2 | 0.55 | 0.314 | 0.93 | 0.446 | 0.47 | 0.321 |  |  |
|  | 0.3 | 0.592 | 0.334 | 0.907 | 0.506 | 0.488 | 0.344 |  |  |
|  | 0.4 | 0.634 | 0.357 | 0.885 | 0.565 | 0.509 | 0.371 |  |  |
|  | 0.5 | 0.673 | 0.383 | 0.858 | 0.622 | 0.53 | 0.395 |  |  |
|  | 0.6 | 0.715 | 0.418 | 0.835 | 0.682 | 0.557 | 0.429 |  |  |
|  | 0.7 | 0.755 | 0.459 | 0.793 | 0.745 | 0.581 | 0.457 |  |  |
|  | 0.8 | 0.794 | 0.513 | 0.739 | 0.809 | 0.606 | 0.487 |  |  |
|  | 0.9 | 0.832 | 0.599 | 0.659 | 0.88 | 0.628 | 0.521 |  |  |
|  | 0.95 | 0.853 | 0.687 | 0.574 | **0.929** | 0.625 | **0.538** |  |  |

**Supplementary table 3.** Performance comparison among different methods.

| **method** | | **Accuracy** | **Precision** | **Recall** | **Specificity** | **F1 score** | **MCC** | **ROC-AUC** | **PR-AUC** |
| --- | --- | --- | --- | --- | --- | --- | --- | --- | --- |
| Semi-supervised GAN | SGAN (1,000 variants) | 0.676 | 0.386 | 0.864 | 0.625 | 0.534 | 0.402 | **0.854** | **0.688** |
|  | SGAN (4,000 variants) | 0.673 | 0.383 | 0.858 | 0.622 | 0.53 | 0.395 | **0.854** | 0.686 |
| Spervised Learning | RF | 0.538 | 0.307 | 0.919 | 0.434 | 0.461 | 0.303 | 0.828 | 0.677 |
|  | SVM | 0.514 | 0.298 | 0.937 | 0.398 | 0.452 | 0.293 | 0.802 | 0.573 |
|  | GBT | 0.544 | 0.302 | 0.86 | 0.457 | 0.447 | 0.267 | 0.795 | 0.566 |
|  | MLP | 0.356 | 0.24 | 0.927 | 0.2 | 0.382 | 0.138 | 0.588 | 0.231 |
|  | VC | 0.483 | 0.28 | 0.897 | 0.37 | 0.427 | 0.236 | 0.816 | 0.674 |
|  | XGB | 0.562 | 0.309 | 0.842 | 0.485 | 0.452 | 0.273 | 0.787 | 0.607 |
|  | ABT | 0.624 | 0.345 | 0.838 | 0.566 | 0.489 | 0.332 | 0.796 | 0.556 |
| function-prediction | SIFT | 0.302 | 0.231 | 0.969 | 0.12 | 0.373 | 0.122 | 0.716 | 0.472 |
|  | PolyPhen2-HDIV | 0.501 | 0.289 | 0.909 | 0.389 | 0.438 | 0.261 | 0.735 | 0.551 |
|  | PolyPhen2-HVAR | 0.545 | 0.304 | 0.867 | 0.458 | 0.45 | 0.273 | 0.75 | 0.507 |
|  | MuTaster | 0.215 | 0.214 | 0.999 | 0.001 | 0.353 | -0.009 | 0.568 | 0.596 |
|  | MuAssessor | 0.346 | 0.231 | 0.882 | 0.2 | 0.367 | 0.088 | 0.634 | 0.297 |
|  | FATHMM | **0.776** | **0.482** | 0.588 | **0.828** | 0.53 | 0.388 | 0.83 | 0.565 |
|  | PROVEAN | 0.558 | 0.282 | 0.686 | 0.523 | 0.4 | 0.172 | 0.648 | 0.311 |
|  | VEST3 | 0.473 | 0.274 | 0.884 | 0.361 | 0.419 | 0.218 | 0.729 | 0.459 |
|  | fitCons | 0.216 | 0.215 | 0.999 | 0.002 | 0.353 | 0.01 | 0.579 | 0.254 |
|  | fitCons (confidence value) | 0.214 | 0.214 | **1** | 0 | 0.353 | 0 | 0.501 | 0.607 |
| ensemble | CADD (raw) | 0.577 | 0.303 | 0.747 | 0.531 | 0.431 | 0.228 | 0.661 | 0.285 |
|  | CADD (phred) | 0.612 | 0.306 | 0.64 | 0.604 | 0.414 | 0.201 | 0.661 | 0.291 |
|  | DANN | 0.241 | 0.219 | 0.99 | 0.037 | 0.359 | 0.064 | 0.578 | 0.233 |
|  | FATHMM-MKL | 0.326 | 0.233 | 0.936 | 0.159 | 0.373 | 0.113 | 0.635 | 0.306 |
|  | MetaSVM | 0.738 | 0.437 | 0.767 | 0.73 | 0.557 | 0.421 | 0.807 | 0.414 |
|  | MetaLR | 0.735 | 0.434 | 0.78 | 0.723 | **0.558** | **0.424** | 0.84 | 0.597 |
| conservation | LRT | 0.24 | 0.217 | 0.975 | 0.04 | 0.355 | 0.031 | 0.566 | 0.541 |
|  | GERP++ | 0.241 | 0.218 | 0.984 | 0.038 | 0.357 | 0.051 | 0.609 | 0.27 |
|  | pP7way (vertebrate) | 0.215 | 0.214 | 0.998 | 0.002 | 0.353 | -0.004 | 0.661 | 0.402 |
|  | pP20way (mammalian) | 0.214 | 0.214 | 1 | 0 | 0.353 | 0 | 0.629 | 0.34 |
|  | pC7way (vertebrate) | 0.377 | 0.242 | 0.896 | 0.235 | 0.381 | 0.133 | 0.647 | 0.403 |
|  | pC20way (mammalian) | 0.383 | 0.245 | 0.903 | 0.241 | 0.386 | 0.145 | 0.657 | 0.426 |
|  | SiPhy29way | 0.593 | 0.237 | 0.406 | 0.643 | 0.299 | 0.042 | 0.562 | 0.233 |

**Supplementary table 4.** Predictive score distribution for loss of function mutations and gain of function mutations.

|  | GoF mutations | LoF mutations |
| --- | --- | --- |
| score < 0.8 | 7 (11.9%) | 62 (4.5%) |
| 0.8 ≤ score < 0.95 | 4 (6.8%) | 542 (39.4%) |
| 0.95 ≤ score | 48 (81.4%) | 771 (56.1%) |
| Total | 59 | 1375 |
